# Supplementary material for: MetaRibo-Seq measures translation in microbiomes
Source: Nat Commun. 2020 Jun 29;11:3268. doi: 10.1038/s41467-020-17081-z (PMC7324362; doi:10.1038/s41467-020-17081-z)
Supplement: Supplementary file 10 — Supplementary Data 7 [file 41467_2020_17081_MOESM10_ESM.zip › File2/Confidence_High_Taxonomy/104550_out.krona.html]

Javascript must be enabled to view this page.

members
magnitude
magnitudeUnassigned
count
unassigned
taxon
rank

104550\_out

3

1

SRS018427\_contig\_number\_14971

2
2
superkingdom

phylum
2
1239

class
2
186801

186802
2
order

1
family

SRS148721\_contig\_number\_51885
2
541000


SRS104400\_contig\_number\_1860
1520815
species
1
